# Nonlinear two-photon pumped vortex lasing based on quasi-bound states in the continuum from perovskite metasurface

**DOI:** 10.1126/sciadv.adf6649

**Published:** 2023-05-31

**Authors:** Chi-Ching Liu, Hui-Hsin Hsiao, Yun-Chorng Chang

**Affiliations:** ^1^ Department of Physics, National Taiwan University, Taipei, Taiwan.; ^2^ Research Center for Applied Sciences, Academia Sinica, Taipei, Taiwan.; ^3^ Nano Science and Technology, Taiwan International Graduate Program, Academia Sinica and National Taiwan University, Taipei, Taiwan.; ^4^ Department of Engineering Science and Ocean Engineering, National Taiwan University, Taipei, Taiwan.

## Abstract

The experimental observation of nonlinear two-photon pumped vortex lasing from perovskite metasurfaces is demonstrated. The vortex lasing beam is based on symmetry-protected quasi-bound states in the continuum (QBICs). The topological charge is estimated to be +1 according to the simulation result. The quality factor and lasing threshold are around 1100 and 4.28 mJ/cm^2^, respectively. Theoretical analysis reveals that the QBIC mode originates from the magnetic dipole mode. The lasing wavelength can be experimentally designed within a broad spectral range by changing the diameter and periodicity of the metasurface. The finite array size effect of QBIC can affect the quality factor of the lasing and be used to modulate the lasing. Results shown in this study can lead to more complex vortex beam lasing from a single chip and previously unidentified ways to obtain ultrafast modulation of the QBIC lasing via the finite array size effect.

## Teaser

Nonlinear pumped vortex lasing based on quasi-bound states in the continuum was observed from the perovskite metasurface.

## INTRODUCTION

Metasurfaces, consisting of two-dimensionally arranged nano-elements, have attracted massive research attention in the past decade due to their unprecedented abilities for light manipulation (*1*–*4*) and led to a variety of ultrathin and lightweight optical devices (*5*–*11*). Compared to plasmonic metasurfaces, the high refractive index dielectric metasurfaces are beneficial to achieve high quality factor (Q factor) resonance owing to the intrinsic low dissipation loss of dielectric materials. The nano-elements of a light-emitting metasurface can absorb the incoming light and re-emit light afterward and the re-emitted light is also molded by the same metasurface (*12*–*14*). When the electromagnetic hot spots overlap with the nano-emitters, the re-emission of photons from the individual nano-element can be substantially increased. In addition, the metasurfaces also functionalize as nanoantennae and affect the far-field radiation pattern of the re-emitted light. However, these emitters are not ideal for laser applications due to the radiation loss.

To substantially suppress the out-of-plane radiative losses and enhance the Q factor of the resonant mode, the optical bound states in the continuum (BICs) have been widely studied in a variety of periodic nanostructures (*15*–*19*). BIC arises from the coupling between the Mie resonance of the nanoantennae and the diffraction mode of the periodic lattice. The radiation channel of the emitters is orthogonal to the BIC resonance, leading to an infinite Q factor when the BIC condition is strictly satisfied. Various types of high Q factor BIC cavities have been demonstrated not only to enhance light-matter interaction but also to achieve lasing action (*20*–*26*) and nonlinear optical phenomena (*27*, *28*). In addition, the vortex property from the emission of symmetry-protected BIC microlasers or nanolasers has been investigated which is promising to be applied as an effective light source for classical or quantum communications (*20*, *26*). For example, a microlaser consisting of periodic nanoholes drilled in perovskite (PVSK) thin film has been demonstrated to exhibit a topological-protected BIC mode with a radial polarization vortex in the far-field radiation (*22*). An all-optical controlled vortex microlaser is achieved by tailoring the highly sensitive property of the symmetry-breaking perturbations of a BIC mode, where the far-field radiation patterns of the laser beams can be modulated by different optical pumping geometry and achieve ultrafast polarization state switching via two-beam pumping (*22*). Arrays of titanium dioxide nanocylinders covered with colloidal CdSe/CdZnS core-shell nanoplatelets were found to support BIC with magnetic dipole (MD) resonant property and achieve optically pumped lasing at room temperature (*26*). The ideal BIC only exists in infinite extended periodic structures under theoretical calculations. In a real system, finite sample size, material absorption, substrate losses, and local defects all limit the Q factor to a finite value, which is typically referred to as quasi-BIC (QBIC) (*15*, *29*, *30*).

PVSK is a novel material that has found huge signs of progress in the fields of solar cells and light-emitting devices within the past decade (*31*–*34*). The high refractive index and strong light-emitting quantum efficiency of PVSK make it an ideal candidate to fabricate emission nanoantennae. The traditional method to synthesize PVSK thin film involves a low-temperature solution process. The resulting thin film is typically polycrystalline film that is composed of tiny nanocrystals (*32*, *35*–*37*). Therefore, it is very difficult to fabricate suitable cavities to sustain laser operation. In addition, the synthesized PVSK thin films are easily damaged when exposed to charged plasma during dry etch or several common solvents and acids during wet etch processes (*38*–*43*). The limitation on the etch process has severely gained difficulty for the realization of PVSK-based nanophotonic devices. In addition, PVSK is also a very good nonlinear material and has been demonstrated to exhibit nonlinear multiphoton absorption and photoluminescence (PL) (*44*–*49*). One-photon (1P) pumped PVSK laser based on symmetry-protected BIC has been reported and the corresponding structures are either one-dimensional (1D) grating or 2D photonic crystal slabs (*22*, *25*). The two-photon (2P) pumped QBIC lasing is challenging because the 2P absorption cross-section is typically several orders of magnitude smaller than the 1P absorption cross section, especially for metasurfaces as thin as 100 nm (*50*).

Here, we have demonstrated the first experimental observation of nonlinear 2P pumped vortex lasing at room temperature from PVSK metasurfaces based on symmetry-protected BIC. The 2P pumped lasing can be more stable because the defects and contamination on the surface of the PVSK metasurfaces affect less to the lasing. The random noise from the laser intensity fluctuation is also notably reduced (*51*). The lasing wavelength can be specifically designed by varying the size and periodicity of the PVSK metasurface. We also investigated the finite size effect of BIC and found that a tiny shift in the sample plane can quickly turn off the QBIC lasing, which can be applied to obtain ultrafast modulation of the BIC lasing.

## RESULTS

The metasurface in this study is a square array of PVSK nanoantennae fabricated on top of a quartz substrate. Electron beam lithography is used to obtain the designed nanopatterns. PVSK materials are subsequently deposited with a vacuum-based two-step process. Detailed information about the process can be found in Materials and Methods. The x-ray diffraction spectra shown in fig. S1 confirm that the precursor PbI_2_ fully transformed to PVSK after the two-step process. The fabricated PVSK nanopattern is covered with a thick film of polystyrene (~600 nm) to form an index-matching environment and also prevents the PVSK nanoantennae from exposing to the air. The thickness of the fabricated PVSK nanoantennae is 100 nm. The diameter and the periodicity of the PVSK nanoantennae can be precisely controlled using the proposed fabrication method. Figure S2 illustrates the scanning electron microscope (SEM) image of the PVSK nanoantennae with various diameters and periodicities. The tilted SEM image shown in fig. S2 also confirms that the height of each PVSK nanoantenna is around 100 nm. The vacuum-based two-step process enables large and uniform PVSK film deposition on top of the structured surface, which is ideal to obtain PVSK nanopatterns.

**Fig. 1.**
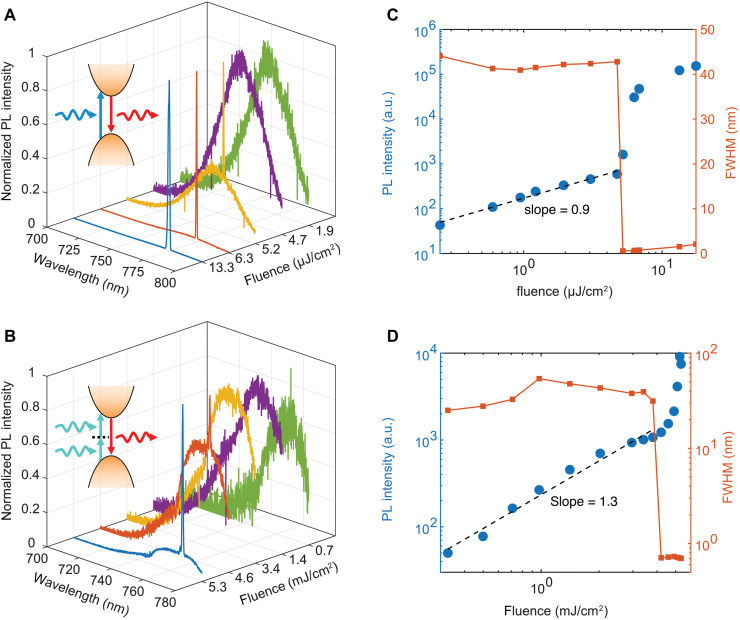
Power-dependent photoluminescence spectra of the PVSK metasurface. (**A** and **B**) One-photon and two-photon pumped PL spectra of the PVSK metasurface at different excitation fluence. Insets in (A) and (B) indicate the corresponding 1P-PL and 2P-PL processes. (**C** and **D**) Corresponding intensity and the full width at half maximum (FWHM) of the PL peak as a function of the excitation fluence. a.u., arbitrary units.

Figure 1 (A and B) illustrates the 1P and 2P pumped PL spectra of the PVSK metasurface, respectively. The wavelength of the excitation laser source is 400 nm for 1P-PL and 800 nm for 2P-PL. The sample consists of a square array of PVSK nanodisk with a periodicity of 475 nm. The diameter and height of the nanodisks are 335 and 100 nm, respectively. In both figures, the PL reveals a broad signal centered near 770 nm and the intensity grows with the increasing excitation power density. Once the excitation is above the threshold power density, a sharp lasing peak appears. The wavelength and full width at half maximum (FWHM) of the 1P lasing peak are 775 and 0.564 nm, respectively. The corresponding Q factor is around 1370. The threshold power density is 5.2 μJ/cm^2^. For 2P lasing, the lasing peak is around 768 nm with a FWHM of 0.7 nm. The Q factor is around 1100. The threshold power density is 4.28 mJ/cm^2^. Figure 1 (C and D) illustrates the variation of the PL peak intensity and its FWHM as a function of the excitation power density. The slope of the light-light curves of the 1P-PL, shown in Fig. 1C, is 0.9, indicating a 1P process. The light-light curve in Fig. 1C also demonstrates two kinks indicating the occurrence of lasing. The slope of the light-light curves of the 2P-PL, shown as the dash lines in Fig. 1D, is around 1.3. This value is larger than one indicating the emission originates from a 2P process. To the best of our understanding, this is the first experimental observation of the 2P pumped QBIC lasing reported in the literature. The deviation from the ideal value of 2 was mainly due to the wavelength of the excitation laser being very close to the bandgap of PVSK and the direct 1P absorption of the laser light can affect the obtained slope, which is commonly seen when determining the nonlinear optical properties of materials (*52*). The light-light curve in Fig. 1D demonstrates only one kink and the laser light was starting to damage the PVSK when the laser fluence was as high as 5.33 mJ/cm^2^. Therefore, it is not possible to demonstrate the second kink. Since the emission peak in the 2P-PL is as narrow as 0.7 nm, which is very close to the linewidth of the 1P lasing peak, the narrow peak shown in 2P-PL should correspond to lasing instead of amplified spontaneous emission.

Note that the difference in lasing wavelength of 1P and 2P lasing is because different samples were used. This is due to the sample was damaged by the laser when the laser power is near the highest during the power-dependent measurement shown in Fig. 1 (C and D). If the same sample was used, then the lasing wavelength difference is not notable, as illustrated in fig. S3. In addition, the linewidth was observed to be more broad with the increasing excitation laser fluence after lasing in Fig. 1C. The broadening could be attributed to the band-filling and thermal broadening, which has been observed in III to V semiconductor lasers (*53*, *54*).

**Fig. 2.**
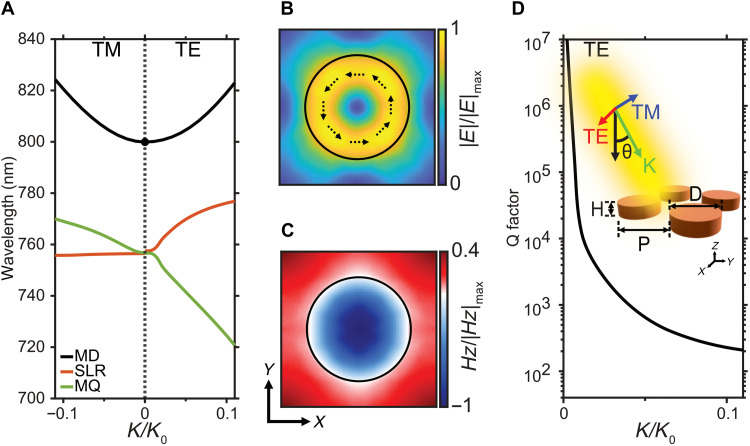
Simulated dispersion relation of the PVSK metasurface. (**A**) Dispersion relation of the PVSK metasurface in both the transverse magnetic (TM) and transverse electric (TE) directions. (**B** and **C**) Corresponding ∣***E***∣ and ***H***_***Z***_ distributions across the sample plane at MD-BIC. Dashed arrows in (B) indicate the direction of the electric field. (**D**) Calculated Q factors for the MD mode in the TE direction. Inset illustrated the schematic view of the PVSK metasurface. MQ, magnetic quadrupole; SLR, surface lattice resonance.

To investigate the origin of the lasing peak, we performed numerical simulations using the finite element method. The diameter and periodicity are set to be 335 and 475 nm, respectively. Figure 2A illustrates the eigenmode solution of the nanoantenna array under transverse electric (TE)– or transverse magnetic (TM)–polarized excitation and both reveal three different modes. Figure S4 illustrates the electric field distributions for these three different modes. The first mode reveals a circular distribution for the electric field with the direction of electric field forms a close loop, as shown in Fig. 2B. This mode is a vertical MD mode shown in Fig. 2C. The second mode reveals a horizontal electric dipole coupled to the incident field. Each nanoantenna oscillates in phase in the *x* direction and becomes a standing wave in the *y* direction. The second mode is referred as the surface lattice resonance mode. The third mode is a high-order resonance, for which four vertical MDs form magnetic quadrupoles (MQs). The electric field mainly concentrated outside the nanoantenna. Figure 2D is the calculated Q factor for the MD mode under the TE-polarized excitation. The Q factor rapidly increases to infinite as the incident angle approach to zero. This result indicates that this MD mode corresponds to a symmetry-protected BIC mode. The vertical dipoles were resonant in phase along the in-plane direction and destructively interfere along the out-of-plane direction. Therefore, no radiation will emit into the free space, resulting in an infinite Q factor. Note that the magnetic field for the MD-BIC mode is strongly confined within the PVSK nanoantenna, which should greatly enhance the PL efficiency and the nonlinear optical absorption cross section.

**Fig. 3.**
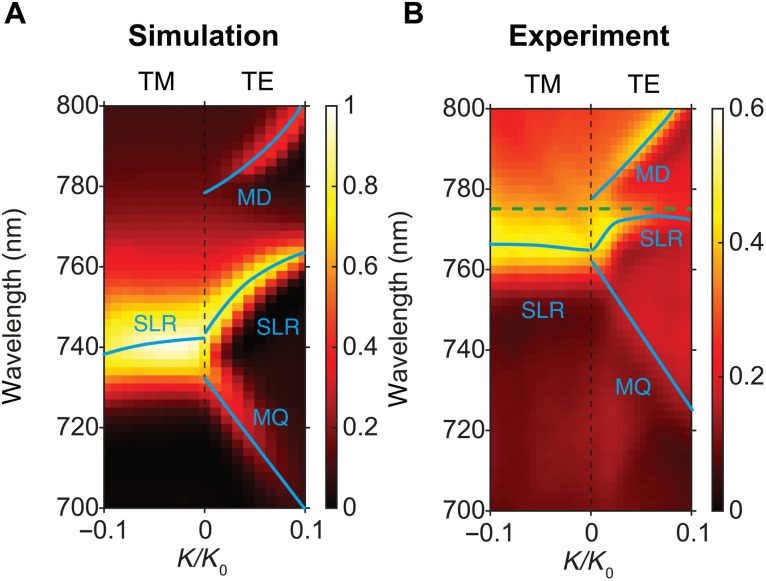
Angle-resolved reflection spectra for the PVSK metasurface. (**A**) Simulated and (**B**) experimental angle-resolved reflection spectra for the PVSK metasurface. Only the SLR mode can be observed when illuminated with TM-polarized light. The green dash line in (B) indicates the observed lasing wavelength.

The eigenmode analysis shown in Fig. 2 indicates that a symmetry-protected BIC mode exists in our fabricated PVSK metasurface. To experimentally prove the existence of this BIC mode, the angle-resolved reflection spectra of the PVSK metasurface were measured. The reflection of a symmetry-protected BIC mode should vanish when the incident angle is at 0° due to the high Q factor, which can be observed in the simulated and experimental reflection spectra shown in Fig. 3 (A and B). The experimental angle-resolved reflection spectra are measured by recording the reflecting light image at the back focal plane (BFP) of the microscope objective [50×, numerical aperture (NA) = 0.55]. The polarization and wavelength of the incident light can be controlled using a linear polarizer and a digitally controlled acousto-optic tunable filter. A gold mirror is used as the reflectivity reference. In the simulated spectra shown in Fig. 3A, three resonance energy bands appear when illuminated with TE-polarized light. Only one band appears when illuminated with TM-polarized light. It is because the MD and MQ modes are generated by vertical MDs. The symmetry feature of MD is disrupted under the angled TE excitation and opens the radiation channel to the space and reduces the Q factors. The angled TM excitation does not interrupt the symmetry of mode and shows high Q factor. Therefore, Q factors of both BIC modes decrease at angled TE field and can be tracked through the spectral method. The experimentally obtained spectra are shown in Fig. 3B, which is consistent with our simulated spectra shown in Fig. 3A. It should also be noted that the gain spectral range of MAPbI_3_ is between 760 and 780 nm, which is very close to the MD mode at a zero-incident angle. Therefore, this lasing mode should correspond to the MD-BIC mode. The green dash line in Fig. 3B indicates the observed lasing wavelength. We also need to point out that the wavelength of the experimental spectra is slightly different compared to the simulated spectra, which is probably due to the variation of the refractive index of surrounding mediums used in the simulation. In addition, the simulation results between Figs. 2A and 3A are slightly different due to the difference in refractive index used in COMSOL. Detailed information is explained in Materials and Methods.

**Fig. 4.**
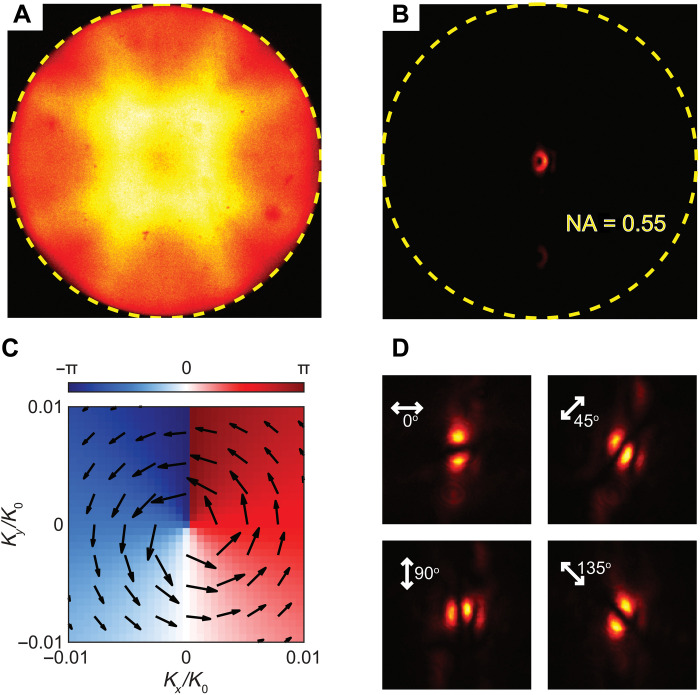
PL distribution at the back focal plane of the microscope objective. Panchromatic BFP PL distribution of the PVSK metasurface when the excitation power is (**A**) below and (**B**) above the lasing threshold. (**C**) Simulated far-field polarization state of emission. The length of the arrow indicates the magnitude of the field. (**D**) Polarization-selected BFP image when the excitation power is above the lasing threshold.

Emission from symmetry-protected BIC lasers is usually a vortex beam, which can be verified by measuring the far-field angular distribution of the emission using the BFP imaging method (*22*–*24*). The excitation laser light is illuminated on the sample through a microscope objective (50×, NA = 0.55) and the PL emission is collected by the same objective. The PL distribution at the BFP of the objective is recorded. Figure 4A is the panchromatic PL image at the BFP of the microscope objective when the excitation is below the lasing threshold. A wide diffraction pattern corresponding to the C4 symmetry is observed, which originates from the diffraction of the PL emission from the square arrays of the PVSK nanoantenna. When the excitation is above the lasing threshold, a donut-shaped emission pattern is clearly observed at the center of the image, shown in Fig. 4B. The dark center is caused by the BIC mode decoupled from the radiation field, which is the signature of the symmetry-protected BIC lasing. The angle of donut-shaped emission is ~2°. This result strongly supports the idea that the observed lasing is from the symmetry-protected BIC mode. Figure 4C is the simulated far-field polarization state of emission when placing a vertical MD inside the center of the PVSK nanoantenna within a square lattice, corresponding to a MD-BIC mode. The arrow points to the direction of the electric field and the length indicates the amplitude. The polarization state is circulating around BIC and forms vortices with a topological charge *q* = +1, calculated from the following equation (*16*)

$$q=\frac{1}{2\pi }\oint_{C}k\;\cdot \nabla_{k}\phi (k)$$

where *C* is a closed loop around the BIC. From this simulation, the donut-shaped emission pattern observed at the BFP changed to two bright lobes when a linear polarizer is inserted before the camera. The orientation of the two lobes will change according to the orientation of the linear polarizer, which is experimentally confirmed from the polarization-selecting BFP image shown in Fig. 4D. When the polarizer is positioned at 0°, two bright lobes align horizontally. The two lobes rotate counterclockwise direction with the counterclockwise rotation of the linear polarizer. These polarization-selecting BFP images further confirm that the lasing peak is a vortex lasing from symmetry-protected MD-BIC with a possible topological charge of +1 derived from simulations. The polarization-selecting BFP images when the excitation power is below the lasing threshold are shown in fig. S7.

**Fig. 5.**
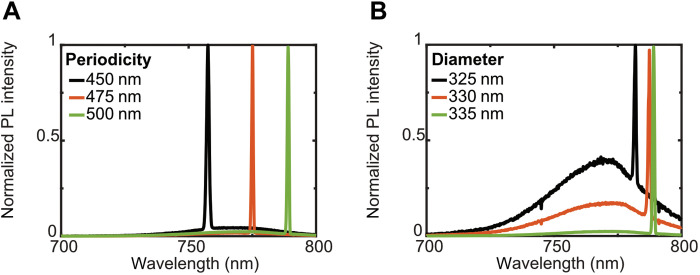
The lasing wavelength with different structural parameters. The lasing wavelength can be designed by changing (**A**) the periodicity of the array and (**B**) the diameter of the nanoantenna.

Since the observed MD-BIC mode originates from the coupling of MD of individual nanoantenna and the surface diffraction, it should be very sensitive to the environment. Therefore, we can easily manipulate the resonant wavelength by changing the periodicity of the arrays or the size of nanoantenna. Figure 5A illustrates the lasing spectra from PVSK nanoantenna arrays with three different periodic lengths. The lasing wavelength of the array with a larger periodicity is observed to shift to a longer wavelength. Figure 5B is the lasing spectra when we vary the diameter of the individual nanoantenna. The periodicity is set at 500 nm. The lasing wavelength is observed to redshift from 782 to 789 nm when the diameter increases from 325 to 335 nm. Since the gain spectra of the PVSK are between 700 to 800 nm, we have plenty of flexibility to obtain BIC lasing within a broad spectra region by carefully selecting the desired combination of the periodicity of the arrays and the size of the nanoantenna. Using the reported method combining both EBL and the two-step method, we can easily adjust the structural parameters of the PVSK metasurface to obtain different resonance wavelengths for various nanophotonic applications.

**Fig. 6.**
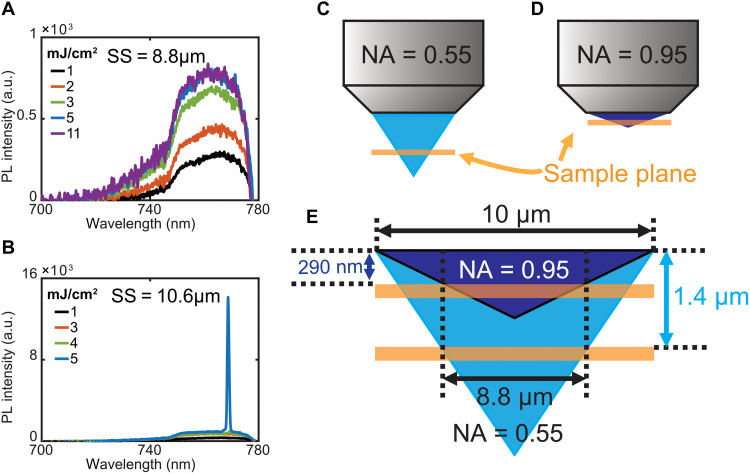
The lasing threshold affected by the finite array size effect. Power-dependent PL spectra of the PVSK metasurface when the diameter of the excitation spot size (SS) is (**A**) 8.8 μm and (**B**) 10.6 μm. Schematic illustration of the light focusing by an objective with (**C**) NA = 0.55 and (**D**) NA = 0.95. The orange bar indicates the sample plane. (**E**) Focusing difference between these two objectives. To reduce the diameter of the illumination area from 10 to 8.8 μm, the sample plane needs to be shifted 1.4 μm vertically for a NA = 0.55 objective. The vertical shift distance is around 290 nm for a NA = 0.95 objective.

Changing the excitation to an asymmetric shape was reported to be able to stop the symmetry-protected BIC lasing without the need to wait the exited carriers to recombine, which typically takes several nanoseconds. The switching time of the modulation can be as fast as 1.5 ps (*22*). In addition to the shape of the excitation spot, the spot size of the excitation is also very crucial for a symmetry-protected BIC lasing. The spot size should cover enough nano-elements to be considered as a “periodic” array that can support BIC. The lasing threshold is reported to be higher when fewer elements are in the array, which is referred to as the finite array size effect of BIC. When performing the 1P-PL measurement using a regular confocal setup, the lasing was not observed when the excitation laser is tightly focused on the sample. The spot size of the laser was around 1.0 μm in diameter. The lasing would appear after we slightly defocus the laser to a spot size of ~23 μm. Since the excitation power density for a tightly focused laser is higher than the defocused laser, the individual PVSK nanoantenna within the laser spot should have enough gain for lasing in both cases. This result strongly confirms that the finite size effect of BIC can be quickly stopped after reducing the excitation spot. The modulation speed of the finite size effect should be as quick compared to the effect from changing the shape of the laser spot since both break the symmetry of a symmetry-protected BIC. The same phenomenon was also observed when we performed 2P-PL measurements and the finite size effect is found to be more prominent for 2P-PL. When the diameter of the laser spot size during 2P-PL is set at 10.6 μm, the lasing peak near 770 nm appears when the excitation power density exceeds 4 mJ/cm^2^, as shown in Fig. 6A. When the diameter of the spot is slightly reduced to 8.8 μm, the lasing peak disappears even if a higher excitation power density is applied, shown in Fig. 6B. The defocus distance of a spot size from 10.6 μm to 8.8 μm is only 1.4 μm using a microscope objective with NA = 0.55, illustrated in Fig. 6C. The defocus distance can quickly drop to ~290 nm if an objective with NA = 0.95 is used, illustrated in Fig. 6D. The comparison is schematically shown in Fig. 6E. These results indicate that the finite array size effect induced by a tiny vertical shift in the sample plane of less than 1 μm can quickly turn off the BIC lasing, which should find previously unknown applications that require ultrafast modulation of the BIC lasing.

**Fig. 7.**
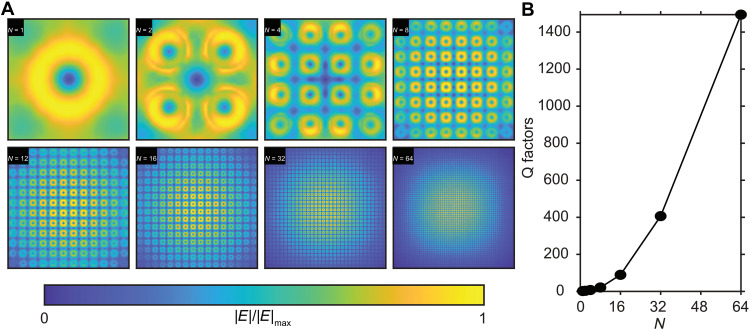
Simulation results explain the finite array size effect. (**A**) Electric field distribution of vertical MD modes at a zero-incident angle for nanoantennas arranged with finite array size (*N* by *N*). (**B**) Calculated Q factors for the MD mode with different array sizes.

To further understand how the finite array size effect can affect the BIC lasing, theoretical simulation was performed to investigate the electric field distribution and Q factors when only a finite array size of *N* by *N* was involved. In the simulation, vertical MD mode of different array sizes at zero incident angle excitation was computed and the results are shown in Fig. 7. Theoretically, MD-mediated BIC is induced via collective resonance of infinite MDs in nanoantennas with coherent phase and intensity. When the laser spot can only excite a limited number of nanoantennas, this deteriorates the resonance integrity of BIC and leads to a finite Q factor. When the array size increases, the mode changes from diffusive to a well-confined electric field within nanoantennas, which mimics the computed field distribution with infinite array size in Fig. 2B. Figure 7B illustrates the corresponding Q factor with different array sizes and the Q factor grows rapidly as an array size increases. This can be attributed to the coherence of MDs that forms QBIC with a reduction of field leakage to the free space. Therefore, the excitation spot size should be large enough to cover a certain number of nanoantennas to support QBIC. The simulation results shown in Fig. 7 clearly explain the finite array size effect. These simulation results agree well with the localized cathodoluminescence mapping reported in the literature (*55*).

In addition, the reported discrete nanoantenna in this study is better than the continuous PVSK film with periodic holes to study the finite array size effect. The carrier diffusion length of a typical PVSK film is around 500 nm, which means that the generated carriers can diffuse within a continuous thin film for half a micrometer (*56*). The carrier diffusion would make the excitation area larger than the actual laser spot, which becomes more apparent when the laser spot is smaller. On the contrary, the carrier cannot diffuse across the discrete nanoantenna arrays proposed in this study so the finite size effect is more evident. The number of nanoantennas involved in the BIC can be precisely counted from the laser spot size.

## DISCUSSION

In summary, the experimental demonstration of 2P pumped vortex lasing is reported in this study. The lasing is based on a symmetry-protected QBIC from PVSK metasurfaces. The Q factor and lasing threshold are around 1100 and 4.28 mJ/cm^2^, respectively. We can also observe 1P pumped vortex lasing and the Q factor and lasing threshold are around 1370 and 5.2 μJ/cm^2^, respectively. The theoretical simulation indicates that the lasing mode corresponds to a MD mode, which is experimentally verified from angle-resolved reflection spectra measured with a BFP imaging method. The emission directionality measurement supports the idea that the lasing is a vortex beam with a topological charge of +1. The lasing wavelength can be designed to a specific wavelength within a broad spectral range by changing the diameter of the nanoantenna and the periodicity. We also study the finite array size effect and discover that a minim excitation spot size is necessary to achieve QBIC lasing. A tiny 1.4-μm vertical shift of the sample plane can quickly turn off the BIC lasing and achieve ultrafast modulation of the lasing. A detailed simulation also confirms the importance of finite array size effect. The 2P pumped QBIC lasing from ultrathin metasurfaces is very challenging because the 2P absorption cross section is typical several orders of magnitude smaller than 1P absorption cross section. The 2P pumped lasing is also more stable and is less affected by surface defects and the fluctuation of laser beams. We believe that these results pave the road for future vortex lasing with the more complicated design of nanoantenna and enable many previously unknown applications in the fields of nanophotonics and quantum optics in the future.

## MATERIALS AND METHODS

### Methylammonium iodide synthetization

HI (57% in water) and methylamine (40% in water) were purchased from Alfa Aesar and used without further purification. Methylammonium iodide (MAI) (CH_3_NH_3_I) was synthesized by reacting the aqueous HI (15 ml) and CH_3_NH_2_ (13.5 ml) at 0°C for 2 hours in a three-neck flask under an N_2_ atmosphere with constant stirring. A white precipitate (CH_3_NH_3_I) formed for 2 hours of rotary evaporation of the solvent. The precipitated white powder was collected, washed three times with diethyl ether, and then dried under a vacuum at 60°C overnight. This dried powder was stored in a glovebox.

### Perovskite nanoantenna fabrication

PVSK nanoantenna arrays were fabricated on quartz substrates. A 300-nm-thick poly(methyl methacrylate) (PMMA; A3 and A5) bilayer was spin-coated on a substrate and patterned by electron beam lithography (Inspect-F, FEI). The bilayer PMMA would form undercut after developing, which is beneficial for following lift-off procedure.

PVSK nanopatterns were subsequently obtained after a vacuum-based two-step process (*57*). First, PbI_2_ (99.9985%, Alfa Aesar) thin film was evaporated onto the pattern substrate in a high vacuum chamber (10^−6^ mTorr, 0.5 ~ 1 Å/s) and the thickness was monitored with a quartz-crystal microbalance. The evaporated PbI_2_ thin film was subsequently loaded into a tube furnace and seated in the center of the furnace. MAI powders were placed 17 cm upstream and the furnace is heated to 120°C to convert PbI_2_ to PVSK. Then the excess PMMA and PVSK were removed in toluene. Last, a thick polystyrene film (~600 nm) was spin-coated on our sample.

### Photoluminescence measuring system

To measure the 1P-PL spectrum, the excitation light source was a tunable pulsed laser with a central wavelength of 400 nm (pulsed width, 100 fs; repetition rate 1, kHz;, Spectra Physics). The laser light was focused on the sample with a 50× microscope objective (NA = 0.55). The PL signal was collected with the same objective and analyzed by a spectrometer equipped with a thermoelectrically cooled charge-coupled device (CCD) camera (Triax 320 and Syncerity, Jobin-Yvon). A 495-nm long-pass filter was placed in front of the entrance of the spectrometer to filter out the excitation laser light. For 2P-PL measurement, the laser wavelength was tuned to 800 nm and an 800-nm short-pass filter was used to remove excitation signals.

### Angle-resolved reflection spectra taken from the back focal plane imaging method

Schematical illustration of the measurement setup is shown in fig. S5A. The sample is illuminated by a linear polarized halogen lamp, which is focused by a 50× objective (NA = 0.55). The reflected light was collected via the same objective. A Fourier lens was inserted so the BFP image of the objective was captured by a 2D electron-multiplying CCD (EMCCD; iXon3, Andor Newton), as shown in fig. S5B. The image was spectrally filtered by a digitally controlled acousto-optic tunable filter (Hsi300, Gooch & Housego) with a 2-nm spectral resolution. TE and TM dispersion surfaces under x-polarized light can be constructed from the K_x_ = 0 and K_y_ = 0 cut, respectively.

### PL emission directionality measurement from back focal plane imaging method

The PL image was generated by shining our sample via a pulsed laser with a central wavelength of 400 nm (pulsed width, 100 fs; repetition rate, 1 kHz; Spectra Physics). The emission BFP image was collected by a 50× objective (NA = 0.55) and focused on a 2D EMCCD (Luca, Andor Newton). A 495-nm long-pass filter was placed before the EMCCD to filter out the excitation laser light, and a linear polarizer was used to probe the radiation polarization state.

### Characterization for the optical constants of the perovskite

The optical constants of the PVSK were determined from spectroscopic ellipsometry measurement (M2000, J. A. Woollam) to model data ranging from 650 to 900 nm for three different incident angles (65°, 70°, and 75°). The fitting was performed on the basis of minimizing the mean square error First, the Cauchy dispersion formula was used to model the transparent region. Subsequently, the B-spline model was used to describe the real and imaginary parts of the optical constant of our PVSK samples. The experimentally obtained optical constants of the PVSK as a function of wavelength are shown in fig. S6.

### Electromagnetic simulations

Band structures, Q factors, reflection spectra, and the corresponding far-field patterns were calculated by a finite-element method using commercial software (COMSOL Multiphysics). We constructed periodic boundary conditions in the *x* and *y* direction and perfectly matched layers along the *z* direction. The optical constant of the PVSK nanoantenna was obtained from the ellipsometry measurement shown in fig. S6. The refractive index of the environment is set to 1.45 to simplify the homogeneous condition. The eigenfrequency solver in COMSOL was used to calculate the eigenstates we are interested in based on the given refractive index values, which is chosen to be the index of PVSK at 790 nm in fig. S6. Eigenfrequency solver was performed under an x-polarized field with a gradual change of parallel k-vector to obtain the band structure and Q factor transition. The TE(TM) field is provided from a sweep in *K_y_*(*K_x_*). In detail, the parallel k-vector is *K_x_* = *n* sin θ*_i_* cos ϕ*_x_* and *K_y_* = *n* sin θ*_i_* sin ϕ*_x_*. Here, *n* is the refractive index of the environment, θ*_i_* is the polar angle incident light from air, and ϕ*_x_* is the azimuthal angle from the *x*-axis. The simulation offers complex eigenfrequency ω = ω*_r_* + *j*ω*_i_* and the corresponding near-field distribution. The dispersion relations are obtained from the real part of ω and the Q factors are given by the ratio *Q* = ω*_r_*/(2 × ω*_i_*), respectively. Frequency domain simulation was performed to calculate the reflection spectra, which allows us to change the refractive index for different wavelengths. The reflection spectra were recorded by impinging a plane wave using ports for excitation and the periodic boundary conditions along the lateral direction. The far-field emission pattern and the polarization vectors were calculated by placing corresponding vertical MDs and vertical electric dipoles in the nanoantenna and sweep in-plane k-vectors.
