# Supplementary material for: Nonlinear two-photon pumped vortex lasing based on quasi-bound states in the continuum from perovskite metasurface
Source: Sci Adv. 2023 May 31;9(22):eadf6649. doi: 10.1126/sciadv.adf6649 (PMC10413678; doi:10.1126/sciadv.adf6649)
Supplement: Supplementary file 1 — Figs. S1 to S7 [file sciadv.adf6649_sm.pdf]

Science Advances
AAAS

Supplementary Materials for

Nonlinear two-photon pumped vortex lasing based on quasi-bound states in the continuum from perovskite metasurface

Chi-Ching Liu *et al*.

Corresponding author: Yun-Chorng Chang, jeffchang@gate.sinica.edu.tw

Sci. Adv. **9**, eadf6649 (2023)
DOI: 10.1126/sciadv.adf6649

This PDF file includes:

Figs. S1 to S7

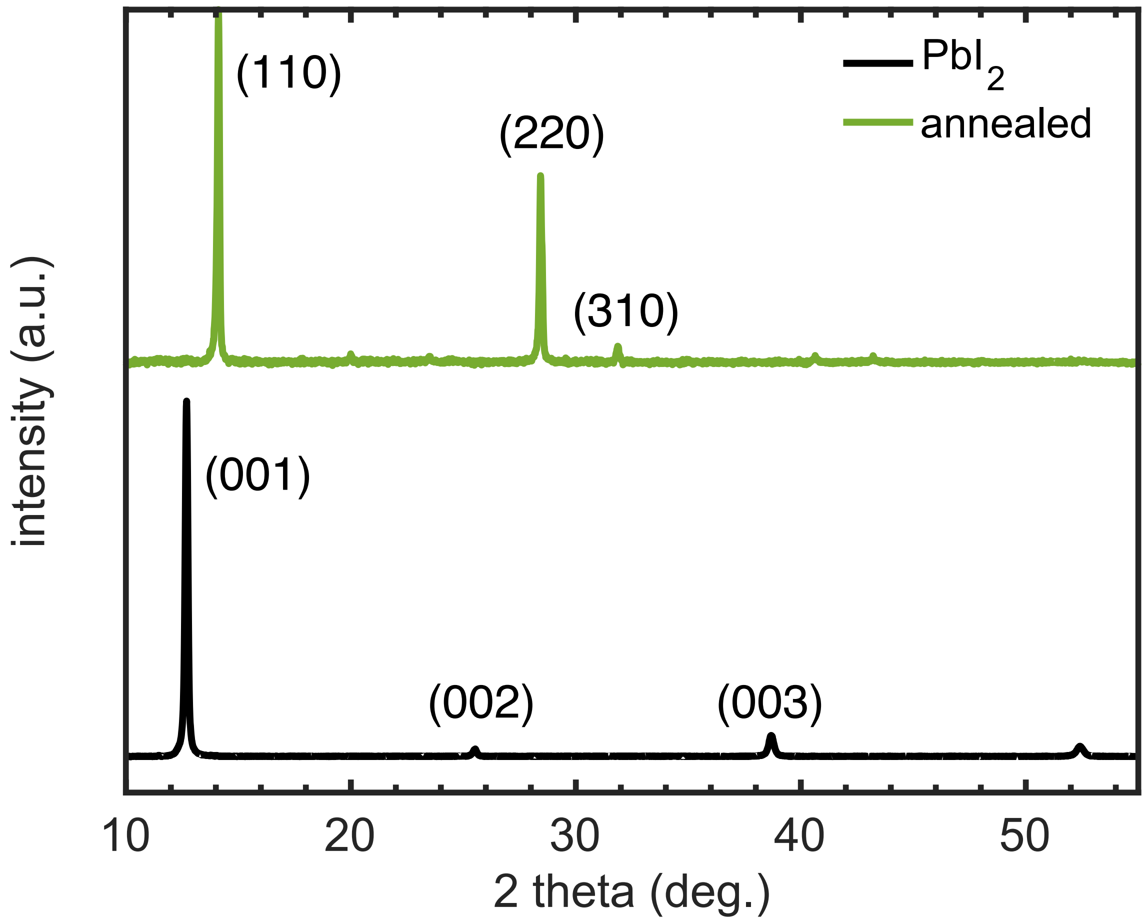

Figure S1. The XRD diffraction spectrum of PbI2, and perovskite films. The XRD diffraction spectrum of PbI_2 and perovskite films prepared with a two-step annealing. After annealing, (110) diffraction peak of perovskite is clearly visible, and (001) peak of PbI_2 becomes vanished, suggesting the film fully is transformed to perovskite.

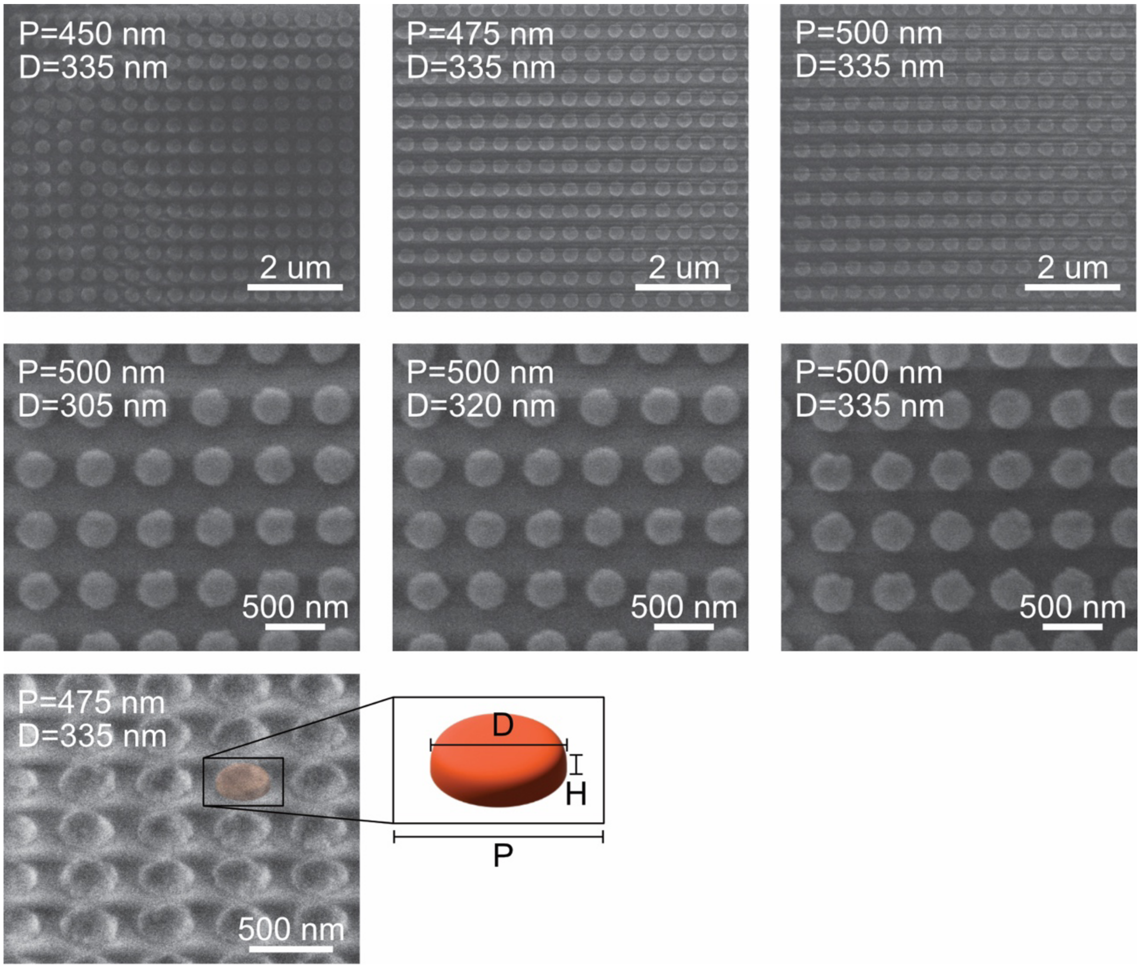

Figure S2. SEM images of perovskite nanoantenna arrays. SEM images of perovskite nanoantenna arrays with different periodicities (P) and diameters (D). The tilted SEM image at the bottom row illustrates the outlook of individual perovskite nanoantenna with a height (H) of 100 nm.

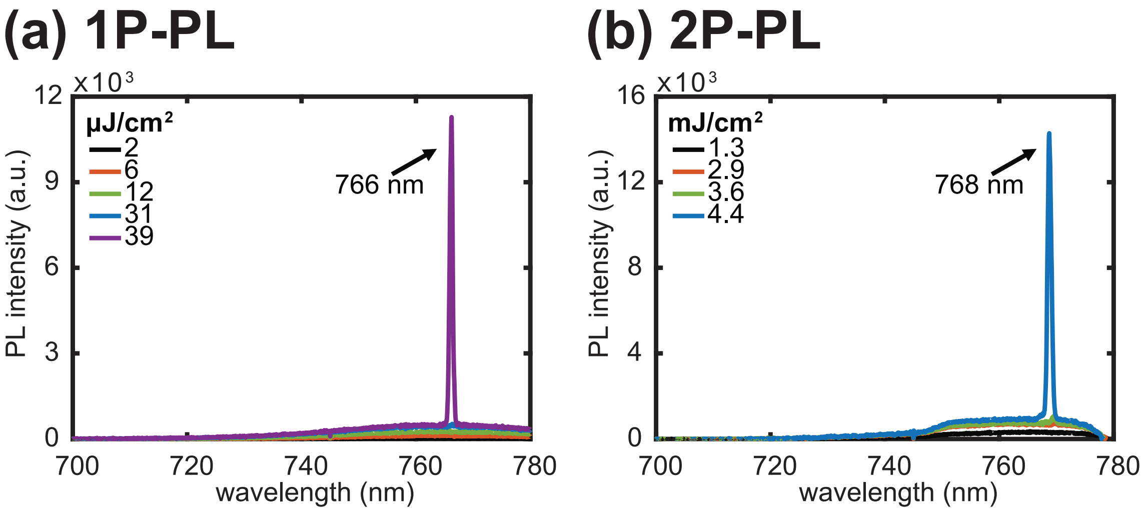

Figure S3. The lasing wavelength comparison between one-photon and two-photon pumped lasing. The lasing spectra of the same sample when excited with (a) one-photon at 400 nm and (b) two-photon at 800 nm. The diameter and height of the nanodisk is 335 nm and 100 nm, respectively. The periodicity of the square lattice is 475 nm. The lasing wavelength slightly red-shifted from 766 nm to 768 nm when changed from one-photon to two-photon pumped.

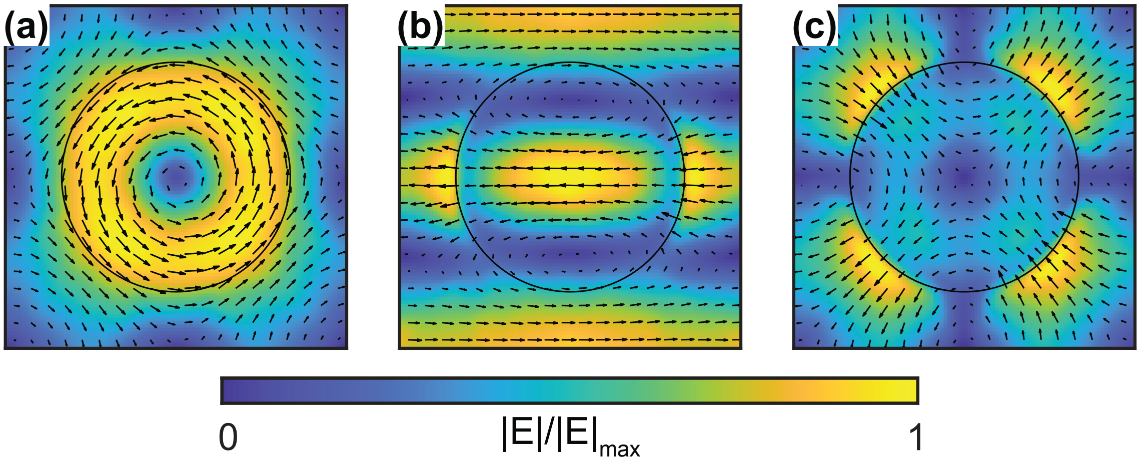

Figure S4. Simulated electric field intensity and polarization vector distributions. Simulated electric field intensity (color map) and polarization vector (arrows) distributions of three eigenmodes of nanoantenna array across sample plane at normal incident angle for (a) MD-BIC, (b) ED-SLR, and (c) MQ-BIC. The length of the arrows also presents the magnitude of field.

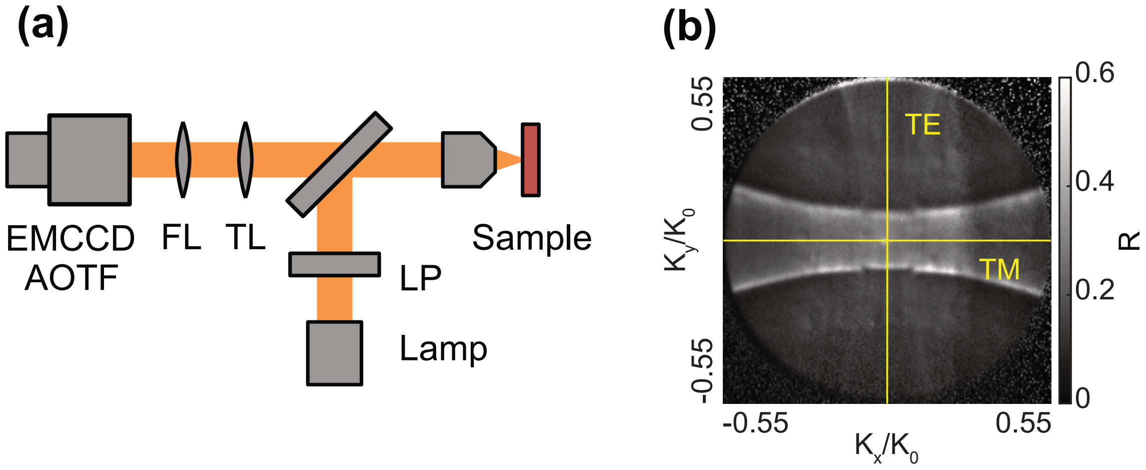

Figure S5. Schematic showing experimental setup and back focal plane reflection image at 800 nm. (a) Schematic experimental setup. The input light passes through a linear polarizer (LP). We image the back focal plane used a tube lens (TL) and fourier lens (FL). AOTF was used to filter the reflected light with a spectral resolution of 2 nm. (b) Back focal plane reflection image at 800 nm. TE and TM dispersion surface under x-polarized light can be constructed from k_x=0 and k_y=0 cut, respectively.

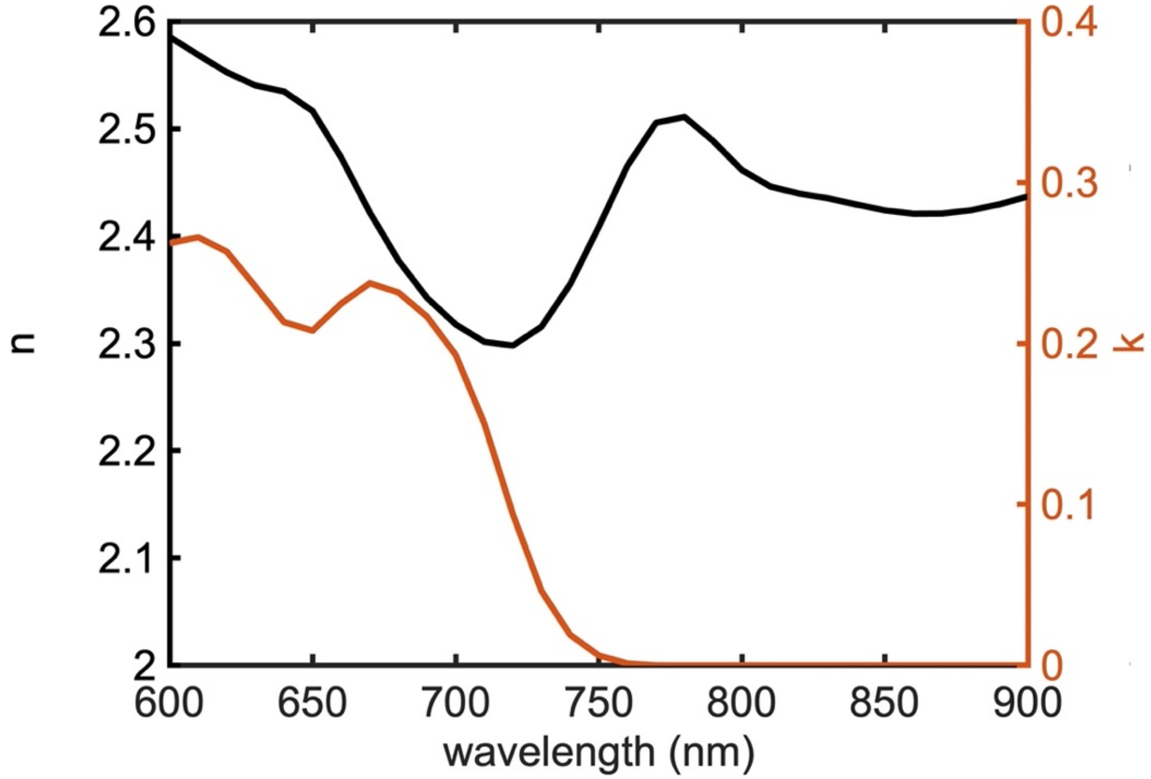

Figure S6. Experimental optical constant of two-step synthesized perovskite thin film. Experimental optical constant of the two-step synthesized perovskite thin film. The real part is above 2.2 close to the emission wavelength of perovskite. The high index value benefits the formation of BIC resonance in nanoantenna arrays. The imaginary part is close to 0 above 760 nm, which is helpful for BIC mediated lasing emission.

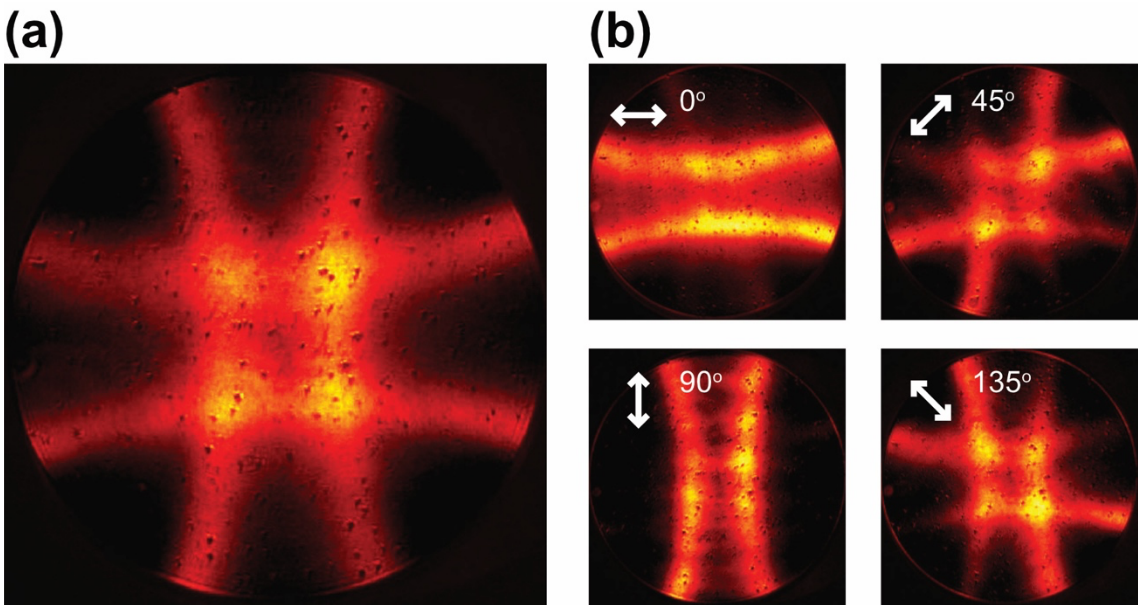

Figure S7. Back focal plane PL distribution when the excitation power is below lasing threshold. BFP PL images of perovskite metasurface when the excitation power is below lasing threshold (a) without using linear polarizer and (b) analyzed using a linear polarizer with various orientations (0°, 45°, 90°, and 135°).
